# Dietary patterns and successful ageing: a systematic review

**DOI:** 10.1007/s00394-015-1123-7

**Published:** 2015-12-22

**Authors:** Catherine M. Milte, Sarah A. McNaughton

**Affiliations:** Centre for Physical Activity and Nutrition Research, School of Exercise and Nutrition Sciences, Deakin University, 221 Burwood Highway, Burwood, Melbourne, VIC 3125 Australia

**Keywords:** Ageing, Diet, Epidemiology, Health, Dietary patterns

## Abstract

**Purpose:**

Nutrition is a key determinant of chronic disease in later life. A systematic review was conducted of studies examining dietary patterns and quality of life, physical function, cognitive function and mental health among older adults.

**Methods:**

Literature searches in MEDLINE complete, Academic Search Complete, CINAHL Complete, Ageline, Global health, PsycINFO, SCOPUS and EMBASE and hand searching from 1980 up to December 2014 yielded 1236 results. Inclusion criteria included dietary pattern assessment via dietary indices or statistical approaches, a sample of community-dwelling adults aged 45 years and over at baseline and a cross-sectional or longitudinal study design. Exclusion criteria included a single 24-h recall of diet, evaluation of single foods or nutrients, clinical or institutionalised samples and intervention studies. Risk of bias was assessed using the six-item Effective Public Health Practice Project’s Quality Assessment Tool for Quantitative Studies.

**Results:**

There were 34 articles (11 cross-sectional and 23 longitudinal) included with 23 studies examining dietary indices and 13 studies using empirical analysis. Most studies examined mental health (*n* = 10) or cognitive function (*n* = 18), with fewer studies examining quality of life (*n* = 6) and physical function (*n* = 8). Although dietary pattern and outcome assessment methods varied, most studies reported positive associations between a healthier diet and better health outcomes.

**Conclusion:**

Overall, the number of studies using dietary patterns to investigate diet and successful ageing is small, and further investigation in longitudinal studies is needed, particularly for quality-of-life outcomes. This review provides support for the importance of a healthy diet for the ageing population globally.

**Electronic supplementary material:**

The online version of this article (doi:10.1007/s00394-015-1123-7) contains supplementary material, which is available to authorized users.

## Introduction

The world’s ageing population continues to increase with the number of older persons expected to exceed the number of children in the world for the first time by 2045 [1]. This will have profound economic implications and influence policies for labour, housing, health care and families. Recognition of the importance that quality of life (QoL) and good overall function accompany this increased life expectancy has led to increased interest in how to promote ‘successful ageing’ [2]. Various definitions of successful ageing exist across social, psychological and medical sciences, but often include life satisfaction and well-being, maintenance of physical and cognitive function and good physical and mental health [2].

The disease burden attributable to chronic disease increases substantially from mid-age; an estimated 80 % of health problems in older age could be prevented or delayed by lifestyle changes in the 55–65 years age group [3]. The most effective programs to support successful ageing would therefore include people from middle age onwards, rather than focussing only on the oldest old or clinical groups [2]. Healthy lifestyle behaviours and fruit and vegetable intake are important factors in the maintenance of QoL in mid-age and older age [4, 5]. Nutrition could therefore play a significant role in health in older age.

Whilst much of the previous research in nutrition and health has focussed on individual nutrients or single foods, there is increasing interest in whole-of-diet analysis, or dietary patterns, as a determinant of chronic disease [6]. Foods contain nutrients and bioactive constituents, which may interact with each other in complex ways. The dietary pattern approach acknowledges that foods are consumed in complex combinations and that balance across the different components of dietary intake may be important. Dietary patterns can be defined by two approaches: multivariate statistical techniques such as factor or cluster analysis (a posteriori, data-driven or empirical approaches) and dietary scoring methods informed by a priori guidelines and recommendations, often referred to as diet quality indices. The a posteriori approaches such as principle component analysis (PCA) or factor analysis create groups by intercorrelated dietary items, whereas cluster analysis groups individuals into patterns based on their reported mean intakes of foods [6]. Diet quality indices can assess adherence to dietary guidelines [7] or to a particular type of diet such as the Mediterranean diet [8]. These measures allow characterisation of diet at a population health level to investigate the impact on health in older populations.

There is evidence that diet quality indices are associated with cardiometabolic risk factors [9], all-cause mortality [10] and lower body physical function [11] in older people. The association between dietary patterns and cardiometabolic outcomes has been investigated in several reviews [12–14]. A recent meta-analysis of dietary patterns and risk of coronary heart disease reported a decreased risk in the highest categories of healthy/prudent dietary patterns and increased risk in the highest categories of the unhealthy/Western-type dietary patterns [15]. There is also growing acceptance that diet may play an important role in brain function and mental health in older age [16, 17]. Recent cross-sectional evidence from Europe implicates diet quality in the incidence of depression, anxiety and cognitive impairment in older adults [18, 19]. Although diet quality has been associated with mortality and increased life expectancy [10], it is also important that older adults have an extended period of stable QoL, free of disability and disease, to enjoy their extended longevity.

A recent umbrella review of food and dietary patterns concluded that there was a limited pool of systematic reviews that followed strict inclusion/exclusion and study quality criteria so that studies are objectively selected and judged and called for more to be undertaken [20]. The purpose of this article is the review literature regarding relationships between dietary patterns and measures of successful ageing in older adults, focusing on QoL, physical function, mental health and cognitive function.

## Methods

### Information sources and search strategy

The current review followed the meta-analysis of observational studies in epidemiology (MOOSE) guidelines [21]. The MEDLINE complete, Academic Search Complete, CINAHL complete, Ageline, Global health, PsycINFO, SCOPUS and EMBASE databases were searched for relevant articles. Search terms were pilot tested to check that appropriate papers were identified before the final search was conducted. The final search included a keyword from each of the following three keyword groups: dietary patterns (diet quality/food patterns/dietary index), older adults (older people/elderly/aged) and health (health status/QoL; physical function/activities of daily living/frailty; mental health/mood/depression; cognitive decline/cognitive performance). The search was limited to publications in English and conducted in humans. As the first papers describing dietary patterns were published in early 1980s [22, 23], the search was restricted to papers published from 1980 onwards. Reference lists of included studies were searched for additional articles. The last search was performed in December 2014.

### Inclusion and exclusion criteria

To be included in this review, studies were required to meet the following criteria: (1) be published as a peer-reviewed original research article, with full-text availability in English; (2) the study participants’ were community-dwelling older adults aged 45 years or older, or aged 45 years or older at study baseline for longitudinal studies; (3) the study reported an assessment of dietary intake including diary records, multiple 24-h recall or Food Frequency Questionnaire (FFQ); (4) the study reported an analysis of the relationship between dietary patterns (including a priori dietary indices or data-driven methods) and an appropriate outcome measure; (5) the study included at least one outcome measure from one of the three areas of interest: health and QoL (self-reported health status), physical function (physical function, activities of daily living, frailty), cognitive function (cognitive decline, cognitive performance) and mental health (depressive symptoms, mood); (6) the statistical analysis included adjustment for relevant covariates.

The exclusion criteria included: (1) the study included only specific or institutionalised populations (e.g. older adults with type 2 diabetes or in residential care); (2) the study combined dietary patterns with other lifestyle/behaviour measures into a ‘lifestyle score’ (except where associations with dietary patterns were reported separately); (3) the study included a single 24-h recall as the dietary assessment method (as this is not reflective of usual intake [24]); (4) the study examined diet from the remote past retrospectively (due to the increased risk of error in dietary recall, which places a greater reliance on memory [24]); (5) the study evaluated single foods or nutrients only; (6) the study included medical records or a self-reported diagnosis for a clinical condition and did not assess current health using an assessment tool or questionnaire.

### Study selection

Titles and abstracts collected from the search were screened by the investigator (CM). Any articles that did not meet eligibility criteria were excluded. For any articles which it was not clear whether they met the eligibility criteria, full-text articles were obtained, screened and resolved by discussion and consensus between the two authors (CM and SM).

### Data extraction and synthesis

Data extraction was assisted by an adapted Microsoft Excel spreadsheet developed for a previous systematic review [25]. Data extracted included: project title, country, study design and aim; inclusion and exclusion criteria; recruitment source; participant age, sex and ethnicity; setting and target populations; time points data collected and reported; measure assessed, measurement tool and unit, measurement tool reliability and validity; total length of follow-up, number of follow-up measurements; total participants enrolled, number of participants included in analyses; number of and/or reasons for withdrawals, dropouts and exclusions, and number lost to follow-up; summary outcome data, type of analysis used, results of analysis; funding source; and key conclusions of authors. Data from the included studies were extracted using a standard form by one author (CM) and verified by a research assistant. In cases of disagreement, discussion was held until consensus was reached. Studies were unable to be combined in a meta-analysis due to the large disparities in methodological approaches, exposures and outcomes.

### Risk of bias assessment

Risk of bias assessment of included articles was conducted using the six-item Effective Public Health Practice Project’s Quality Assessment Tool for Quantitative Studies (see http://www.ephpp.ca/PDF/Quality%20Assessment%20Tool_2010_2.pdf) [26]. This tool considers selection bias, study design, confounders, blinding, data collection methods and withdrawals and dropouts. One author (CM) assessed each of the six risks of bias indicators as ‘low’, ‘medium’ or ‘high’. Studies were then given a global risk of bias rating of ‘low’ (four low ratings and no high ratings), ‘medium’ (less than four low ratings and one high rating) or ‘high’ (two or more high ratings).

## Results

**Fig. 1:**
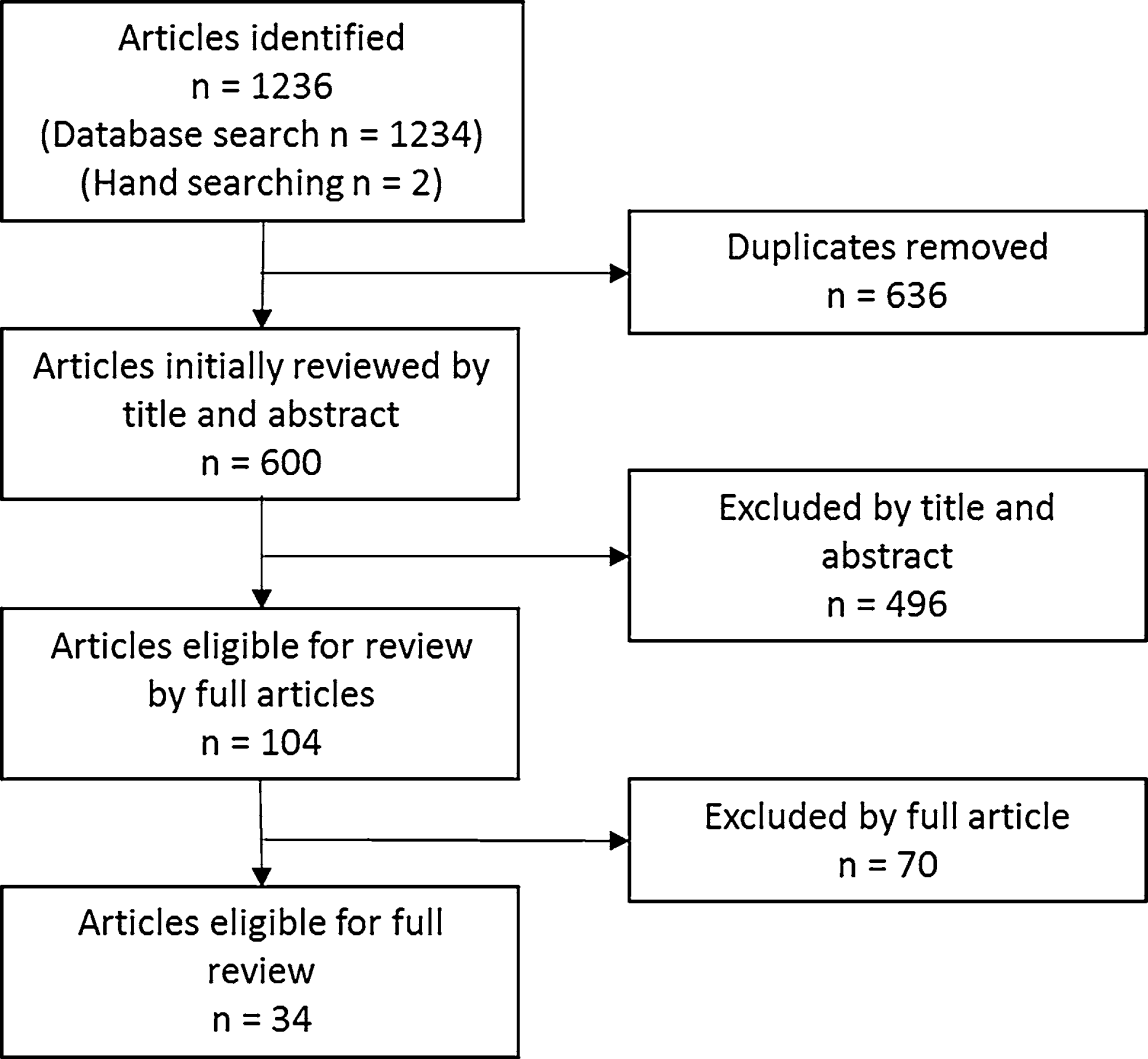
Flow chart summary of articles identified in search and included in review

The search of databases yielded 1234 results, and hand searching yielded an additional two studies (Fig. 1). A total of 636 duplicates were removed, leaving a total of 600 articles to screen titles and abstracts, with 496 articles excluded at this stage. Full-text articles were retrieved and assessed for eligibility (*n* = 104), and 34 were included in the review. Articles were divided into two groups by the whole-diet assessment method used: either a priori dietary indices (Table 1) or data-driven methods (Table 2). Following this, articles were classified into groups according to the outcome measure they assessed: QoL, physical function, mental health and cognitive function.

**Table 1:** Characteristics of selected studies on dietary indices and measures of successful ageing

Reference, country, study name	Design (CS/P/L)	Sample size (% women)	Participants; age, years (mean ± SD)	Follow-up (years)	Dietary intake assessment, index	Outcome	Adjustments^a^	Results	Risk of bias^b^
*Self-rated health and quality of life*									
Ford et al. [27], USA, GRAS	CS	4009 (57 %)	>74 (81.5 ± 4.4)	N/A	Dietary screening tool	HALex	A, G, H, S, self- versus proxy-report	HALex scores were significantly lower for ‘unhealthy’ and ‘borderline’ dietary scores compared to ‘healthy’ dietary score	Mod
Gopinath et al. [28], Australia, BMES	P, L	1305 (59.2 %)	>55 (mean ~67)	5	145-item FFQ, modified Australian DQI	SF-36	A, G, S, H	DQI positively associated with vitality, physical function, physical function and physical composite score	Mod
Woo et al. [29], Hong Kong, N/A	CS	3611 (51.2 %)	≥65 (72.52 ± 5.21)	N/A	266-item FFQ, DQI-I	SF-12 (MCS and PCS)	A, G	DQI-I associated with mental and physical health status	High
Kimura et al. [30], Japan, N/A	CS	689 (58 %)	≥65 (75.7 ± 7.3)	N/A	Food diversity: FDSK-11	QoL visual analogue scale	A	High food diversity group reported higher QoL on subjective health, family relationship, friend relationship and subjective happiness	High
Haveman-Nies et al. [31], Europe, SENECA	L	480 (55 %)	70–75 (men 72.6 ± 1.6, women 72.7 ± 1.7)	10	Modified DH, MDS	Self-rated health status	A, S	No associations between diet quality and health status	High
*Frailty/physical function*									
Shikany et al. [35], USA, MrOS	P, L	5922 (0 %)	Men ≥65 (mean ~75)	4.6	69-item FFQ, DQI-R	CHS Frailty Index	A, Race, S, H, E	DQI-R was inversely associated with frailty status at baseline and follow-up	Mod
León-Muñoz et al. [36], Spain, Seniors-ENRICA	P, L	1815 (~60 %)	≥60 (mean ~68.5)	3.5	DH, MEDAS and MDS	CHS Frailty Index	A, G, S, H, E	Highest MEDAS score tertile associated with lower risk of frailty compared to lowest tertile. MDS associated with progressively reduced risk of frailty	Mod
Gopinath et al. [28], Australia, BMES	P, L	895 (58.2 %)	>55 (mean ~71)	5	145-item FFQ, Modified Australian DQI	The Older American Resources and Services ADL scale	A, G, S, H	Risk of incident IADL at follow-up lowest in highest quartile baseline total diet score	Mod
Bollwein et al. [37], Germany, N/A	CS	192 (64 %)	≥75 (83 ± 4)	N/A	103-item FFQ, MED score	CHS Frailty Index	A, G, S, H, E	Risk of being frail reduced in highest quartile of MED score. Linear trend in OR, indicative of a graded effect for diet. Lower MED score associated with weight loss, low physical activity and low walking speed	High
Milaneschi et al. [11], Italy, InCHIANTI	P, L	935 (55.6 %)	≥65 (74.1 ± 6.8)	9	47-item FFQ, MDS	SPPB	A, G, E, H	Higher MDS associated with better function, less decline in SPPB score and lower risk of developing new mobility disability	Mod
Woo et al. [29], Hong Kong, N/A	CS	3611 (51.2 %)	≥65 (72.52 ± 5.21)	N/A	266-item FFQ, DQI-I	Frailty index	A, G	DQI-I associated with frailty	High
Kimura et al. [30], Japan, N/A	CS	689 (58 %)	≥65 (75.7 ± 7.3)	N/A	Food diversity: FDSK-11	Basic ADL and advanced ADL on Tokyo Metropolitan Institute of Gerontology Index of Competence rating scale	A	High food diversity group reported better basic ADL and better advanced ADL	High
Haveman-Nies et al. [31], Europe, SENECA	L	480 (55 %)	70–75 (men 72.6 ± 1.6, women 72.7 ± 1.7)	10	Modified DH, MDS	Self-care ability	A, S	No associations between diet quality and self-care ability observed	High
*Cognitive function*									
Wengreen et al. [52], USA, CCMS	P, L	3580 (57.1 %)	≥65 (mean ~74)	11	142-item FFQ, DASH and MDS	3MS	A, G, S, H	Higher DASH and MDS associated with higher levels of cognitive function over 11 years	Low
Chan et al. [53], Hong Kong, N/A	CS	3670 (47.5 %)	≥65	N/A	280-item FFQ, MDS	CSI-D	A, H, E, S	No association with MDS and risk of cognitive impairment in men or women	Mod
Katsiardanis et al. [54], Greece, Velestino Study	CS	557 (57 %)	≥65	N/A	157-item FFQ, MedDietScore	MMSE	A, S, H	MedDietScore positively associated with MMSE in men. MedDietScore negatively associated with MMSE in women	Mod
Kesse-Guyot et al. [55], France, SU.VI.MAX	P, L	3083 (46.3 %)	≥45 (52.0 ± 4.6)	13	6 × 1-day DR, MDS and MSDPS	Global cognitive function (R1-48 cued recall (episodic memory), 2 × verbal fluency tasks (lexical semantic memory), forward and backward digit span (short-term and working memory), Delis–Kaplan trail-making test (metal flexibility)	A, G, S, H, E, I, follow-up time, number of FR	No association with global cognitive function. Minor associations between MDS and digit span and MSDPS and phonemic fluency performance	High
Ye et al. [56], USA, Boston Puerto Rican Health Study	CS	1269 (% women not reported)	Puerto Rican 45–75 (57.3 ± 7.6)	N/A	FFQ, MDS and HEI 2005	Cognitive function (neuropsychological test battery and MMSE) and presence of cognitive impairment	A, G, S, H	Higher MDS and HEI 2005 associated with higher MMSE and lower odds of cognitive impairment	Mod
Shatenstein et al. [57], Canada, Nu Age	P, L	1488 (52.6 %)	67–84 (men 74.05 ± 4.09, women 74.36 ± 4.21)	3	78-item FFQ, C-HEI	3MS	A, G, S, H	Overall C-HEI score and some subscores associated with baseline 3MS in univariate analysis. C-HEI not associated with cognition in multivariate analysis	Mod
Vercambre et al. [58], USA, WACS	L	2504 (100 %)	Women ≥65 (range 66.1–91.2) with prevalent CVD or risk factors	5.4	116-item FFQ, MDS	Cognitive function (TICS, East Boston Memory Test, category fluency)	A, S, E, H, I	MDS not related to cognitive decline	Mod
Tangney et al. [59], USA, CHAP	P, L	3790 (61.7 %)	≥65 (75.4 ± 6.2)	7.6	Modified Harvard 139-item FFQ, MedDiet and HEI-2005	Global cognitive function (East Boston immediate and delayed recall; MMSE; symbol digit and modalities test)	A, G, S, cognitive activity, E (for MedDiet)	Med Diet associated with slower rate of cognitive decline. No association with HEI-2005	Mod
Feart et al. [60], France, Three-City Study	P, L	1410 (63 %)	≥65 (67.7–94.9)	5	FFQ, MDS	Cognitive function (MMSE, Isaacs Set Test, Benton Visual Retention Test, Free and Cued Selective Reminding Test)	S, E, H, APOE genotype	Higher MDS associated with fewer MMSE errors. No other associations in the whole cohort. In individuals without dementia over the 5 years, higher MDS was associated with better performance on the MMSE and Free and Cued Selective Reminding Test	Mod
Wengreen et al. [61], USA, CCMS	P, L	3634 (55–59 %)	≥65 (mean ~74)	11	142-item FFQ, RFS and Non-RFS	3MS	A, S, G, APOE genotype, H, E	Higher RFS associated with higher 3MS at baseline, and less decline in 3MS scores over 11 y. No relationship with Non-RFS	Mod
Nicolas et al. [62], France, Toulouse Aging Process Study	P, L	96 (76 %)	≥55 (76.2 ± 5.9)	4	3-day DR, HDI	Cognitive function (MMSE, WAIS symbol digit modalities task, timed cancellation task)	None^c^	Higher HDI in 1993 associated with better MMSE and timed cancellation task. No association with current HDI and cognitive function	High
Huijbregts et al. [63], Europe, Seven Countries Study	CS	1049 (0 %)	Men 70–91 (mean ~76)	N/A	DH, HDI	MMSE	A, S, H, E	A higher HDI score associated with reduced odds of cognitive impairment (statistically significant in Crevalcore cohort only)	Mod
*Mental health*									
Skarupski et al. [53], USA, CHAP	P, L	3502 (59 %)	≥65 (73.5 ± 6.1)	7.2	69-item FFQ, MedDietScore	CES-D	A, G, Race, S, E, H	MedDietScore inversely associated with risk of developing depressive symptoms	Mod
Hodge et al. [43], Australia, MCCS	P, L	8660 (~61.8 %)	50–69 (mean ~59)	12	121-item FFQ, MDS	K10	A, G, S, H, E	MDS inversely associated with psychological distress. Australian-style pattern also inversely associated with psychological distress	Mod
Jacka et al. [18], Norway, Hordaland Health Study	CS	5731 (56.8 %)	46–49 and 70–74	N/A	169-item FFQ, a priori diet quality score	Hospital Anxiety and Depression Scale	A, S, H, E	Diet quality was inversely related to depression in men. Diet quality was inversely related to depression and anxiety n women	Mod
Kimura et al. [30], Japan, N/A	CS	689 (58 %)	≥65 (75.7 ± 7.3)	N/A	Food diversity: FDSK-11	GDS-15	A	High food diversity group reported lower depression scores (4.1 + 3.8 vs. 6.2 + 4.3), *p* < .001)	High

*ADL* activities of daily living, *BMES* The Blue Mountains Eye Study, *CES-D* Centre for Epidemiologic Studies-Depression Scale, *CCMS* Cache Country Study on Memory and Ageing in Utah, *CHAP* Chicago Health and Aging Project, *C-HEI* Canadian Healthy Eating Index, *CHS* Cardiovascular Health Study, *CS* Cross-Sectional, *CSI-D* Community Screening Instrument for Dementia, *DASH* Dietary Approaches to Stop Hypertension, *DH* Diet History, *DQI-I* Diet Quality Index-International, *DQI-R* Diet Quality Index-Revised, *DR* Diet Records, *FDSK-11* 11-item Food Diversity Score Kyoto, *FFQ* Food Frequency Questionnaire, *GDS* Geriatric Depression Scale, *HALex* Health and Activity Limitation Index, *HDI* Healthy Diet Indicator, *HEI 2005* Healthy Eating Index 2005, *K10* Kessler Psychological Distress Scale, *L* longitudinal, *MCS* Mental Component Summary, *MCCS* Melbourne Collaborative Cohort Study, *MDS* Mediterranean Diet Score, *MedDietScore* Mediterranean Diet Score, *MED score* Alternate Mediterranean Food Score, *MEDAS* Mediterranean Diet Adherence Screener, *MrOS* Osteoporotic Fractures in Men, *MSDPS* Mediterranean-Style Dietary Pattern Score, *MMSE* Mini–Mental State Examination, *P* prospective, *QoL* quality of life, *RFS* Recommended Food Score, *SD* standard deviation, *SENECA* Survey in Europe on Nutrition and the Elderly, *SF-12* 12-item Short-Form Health Survey, *SPPB* Short Physical Performance Battery, *SU.VI.MAX 2* Supplémentation en Vitamines et Minéraux 2 study, *TICS* Telephone Interview of Cognitive Status, *WACS* Women’s Antioxidant Cardiovascular Study, *WAIS* Wechsler Adult Intelligence Scale, *3MS* Modified Mini–Mental State Examination

^a^Adjusted for: A, age; G, gender; S, sociodemographics; H, health-related variables; E, total energy intake; I, intervention group

^b^Assessed by Effective Public Health Project Quality Assessment Tool for Quantitative Studies: Low, low risk of bias; Mod, moderate risk of bias; High, high risk of bias

^c^A, S and E were investigated; however, no adjustments used due to lack of consistent effects

**Table 2:** Characteristics of selected studies on data-driven methods and successful ageing

Reference, country, study name	Design (CS/P/L)	Sample size (% women)	Age, years (mean ± SD)	Follow-up (years)	Diet intake assessment, pattern analysis (input)	Outcome	Adjustments^b^	Results	Risk of bias^c^
*Self-rated health and quality of life*									
Samieri et al. [19], France, Three-City Study	CS	1724 (62 %)	≥65 (76.8 ± 5.10)	N/A	148-item FFQ, cluster analysis (20 food groups)	Self-rated health	A, S	Men: 5 clusters ‘small eaters’, ‘biscuits and snacking’, ‘healthy’, ‘charcuterie, meat, alcohol’, ‘pasta eaters’. ‘Pasta eaters’ more likely to report impaired health. Women: 5 clusters ‘small eaters’, ‘biscuits and snacking’, ‘healthy’, ‘charcuterie, starchy foods’, ‘pizza, sandwich’. ‘Biscuits and snacking’ more likely to report impaired health	Low
*Cognitive function*									
Parrott et al. [64], Canada, The NuAge Study	P, L	1099 (49.4 %)	68–84 (mean ~74)	3	78-item FFQ, PCA (78 foods)	3MS	A, G, H, S, E	Two patterns: ‘prudent’ and ‘western’. Prudent pattern associated with higher 3MS scores at recruitment in the upper categories of income, education, or composite SEP, and with less cognitive decline for low composite SEP only. Western pattern associated with greater cognitive decline for low educational attainment only	Mod
Kesse-Guyot et al. [65], France, SU.VI.MAX 2	P, L	2983 (N/A)	45–60 (65.5 ± 45 at follow-up)	13.6	Multiple 24-h DR (bimonthly), RRR (30 food groups)	Global cognitive function (R1-48 cued recall (episodic memory), 2 × verbal fluency tasks (lexical semantic memory), forward and backward digit span (short-term and working memory), Delis–Kaplan trail-making test (metal flexibility)	A, G, H, I, S, E, time	One pattern: ‘carotenoid-rich dietary pattern’. Dietary pattern positively associated with composite cognitive score, and scores for the cued recall task, backward digit span task, trail-making test and fluency task	Mod
Corley et al. [66], UK, Lothian Birth Cohort 1936	CS	878 (~50 %)	~70 (69.5 ± 0.8)	N/A	168-item FFQ, PCA (168 foods)	Moray House Test (age 11 and 70), WAIS cognitive subtests, MMSE	A, G, S	Four patterns: ‘Mediterranean-style’, ‘health aware’, ‘traditional’ and ‘sweet foods’. Mediterranean and traditional patterns associated with better cognitive performance at old age. After adjusting for childhood IQ and SEP, statistical significance was lost for most associations excluding verbal ability for both patterns	High
Chan et al. [53], Hong Kong, N/A	CS	3670 (47.5 %)	≥65	N/A	280-item FFQ, factor analysis (32 food groups)	CSI-D	A, H, E, S	Three patterns: ‘vegetable–fruits’, ‘snacks–drinks–milk products’ and ‘meat–fish’. Men: No dietary patterns associated with risk of cognitive impairment. Women: ‘vegetable–fruits’ and ‘snacks–drinks–milk products’ patterns associated with reduced risk of cognitive impairment	Mod
Kesse-Guyot et al. [67], France, SU.VI.MAX 2	P, L	3054 (~45 %)	≥45 years	13	6 × 1-day DR over 2 years, PCA (34 food groups)	Cognitive function: language and verbal memory (R1-48 cued recall, verbal fluency tasks), executive functioning (forward and backward digit span, Delis-Kaplan trail-making test)	A, G, S, I, E, H,	2 patterns: ‘healthy’, ‘traditional’ Healthy pattern associated with better cognitive function and verbal memory	High
Akbaraly et al. [68], UK, Whitehall II	CS	4693 (26.2 %)	White European ≥52 (mean ~61)	N/A	127-item FFQ, PCA (37 food groups)	Cognitive function (20-word free-recall test of short-term verbal memory, AH4-I verbal and mathematical reasoning, Mill Hill Vocabulary test, phonemic verbal fluency, semantic verbal fluency)	A, G, S, E, H	Two patterns: ‘whole food’ and ‘processed food’. Whole food diet associated with lower odds of cognitive deficit on all five tests. Processed food diet associated with higher odds of cognitive deficit for all tests except memory	Mod
Samieri et al. [19], France, Three-City Study	CS	1724 (62 %)	≥65 (76.0 ± 4.97)	N/A	148-item FFQ, cluster analysis (20 food groups)	MMSE	A, S	Men: 5 clusters ‘small eaters’, ‘biscuits and snacking’, ‘healthy’, ‘charcuterie, meat, alcohol’ and ‘pasta eaters’. Women: 5 clusters ‘small eaters’, ‘biscuits and snacking’, ‘healthy’, charcuterie, starchy foods’ and ‘pizza sandwich’. Lower number of errors on MMSE in ‘healthy’ cluster in both sexes	Low
*Mental health*									
Chan et al. [44], Hong Kong, N/A	P, L	2902 (~40 %)	≥65 (mean ~72)	4	280-item FFQ, factor analysis (32 food groups)	GDS	A, G, E, H, S	Three patterns: ‘vegetable–fruits’, ‘snacks–drinks–milk products’ and ‘meat–fish’. Inverse association with GDS and the ‘vegetable–fruits’ and ‘snacks–drinks–milk products’ patterns at baseline. No association with dietary patterns at 4 years	Mod
Hodge et al. [43], Australia, MCCS	P, L	8660 (~61.8 %)	50–69 (mean ~59)	12	121-item FFQ, PCA	K10	A, G, S, H, E	Two patterns: ‘modified Mediterranean’ and ‘Australian’. Australian-style pattern inversely associated with psychological distress	Mod
Rienks et al. [45], Australia, ALSWH	P, L	8369 CS, 7588 L (100 %)	Women 50–55 (52.5 ± 1.5)	3	101-item FFQ, factor analysis (101 food groups)	CES-D (2001 and 2004)	S, E, H	6 patterns: ‘cooked vegetables’, ‘fruit’, ‘Mediterranean-style’, ‘meat and processed meat’, ‘dairy’ and ‘high fat and sugar’. Mediterranean diet associated with lower depressive symptoms (NS after controlling for confounders)	Low
Le Port et al. [46], France, GAZEL	P, L	12,404 (25 %)	45–60 (men 45 ± 2.9, women 42.2 ± 4.2)	10	35-item FFQ, PCA (35 foods)	CES-D	A, S, H	Men: 5 patterns ‘low fat’, ‘healthy diet’, ‘western diet’, ‘fat-sweet’ and ‘high snacking’. Healthy diet associated with reduced likelihood of depressive symptoms. Low fat, western, fat-sweet and snacking diets were associated with increased likelihood of depressive symptoms. Women: 6 patterns ‘low fat’, ‘healthy diet’, ‘traditional diet’, ‘animal protein pattern’, ‘high dessert’ and ‘high snacking’. Healthy diet and traditional diet associated with reduced likelihood of depressive symptoms. Low fat and snacking diets were associated with increased likelihood of depressive symptoms	Mod
Jacka et al. [18], Norway, Hordaland Health Study	CS	5731 (56.8 %)	46–49 and 70–74	N/A	169-item FFQ, PCA	Hospital Anxiety and Depression Scale	A, S, H, E	Three patterns: ‘healthy’, ‘western’ and ‘traditional (Norwegian)’ Men: A traditional diet was associated with less likelihood of depression. A healthy diet was associated with increased likelihood of anxiety. Women: A healthy diet was associated with less likelihood of depression. A healthy diet and traditional diet were associated with reduced likelihood of anxiety	Mod
Akbaraly et al. [47], UK, Whitehall II	P	3486 (26.2 %)	White European ≥47 (mean 55.6)	5	127-item FFQ, PCA (37 food groups)	CES-D	A, G, S, E, H	Two patterns: ‘whole food’ and ‘processed food’. Intake of whole food pattern associated with lower odds of depression. Intake of processed food pattern associated with increased depression	Mod
Samieri et al. [19], France, Three-City Study	CS	1724 (62 %)	≥65 (76.0 ± 4.97)	N/A	148-item FFQ, cluster analysis (20 food groups)	CES-D	A, S	Men: 5 clusters ‘small eaters’, ‘biscuits and snacking’, ‘healthy’, ‘charcuterie, meat, alcohol’ and ‘pasta eaters’. CES-D scores higher in ‘pasta eaters’ cluster. Women: 5 clusters ‘small eaters’, ‘biscuits and snacking’, ‘healthy’, charcuterie, starchy foods’ and ‘pizza sandwich’. No significant relationships in adjusted model	Low

There were no studies examining frailty or physical function

*ALSWH* Australian Longitudinal Study on Women’s Health, *CES-D* Centre for Epidemiologic Studies-Depression Scale, *CS* cross-sectional, *CSI-D* Community Screening Instrument for Dementia, *DR* diet record, *FFQ* Food Frequency Questionnaire, *GDS* Geriatric Depression Scale, *K10* Kessler Psychological Distress Scale, *L* longitudinal, *MCCS* Melbourne Collaborative Cohort Study, *MDS* Mediterranean Diet Score, *MMSE* Mini–Mental state Examination, *P* prospective, *PCA* principle component analysis, *RRR* reduced rank regression, *SD* standard deviation, *SEP* socioeconomic position, *SU.VI.MAX 2* Supplémentation en Vitamines et Minéraux 2 study, *WAIS* Wechsler Adult Intelligence Scale, *3MS* Modified Mini–Mental State Examination

^a^Adjusted for: A, age; G, gender; S, sociodemographics; H, health-related variables; E, total energy intake; I, intervention group

^b^Assessed by Effective Public Health Project Quality Assessment Tool for Quantitative Studies: Low, low risk of bias; Mod, moderate risk of bias; High, high risk of bias

There were six studies that examined QoL or health status [19, 27–31], which was assessed by either a self-reported single item (with participants asked to rate their present health status as very good, good, fair, bad or very bad), a 5-item 100-mm visual analogue scale [32], or questionnaire-based measures of health-related QoL such as the well-known 12- or 36-item Short-Form Health Survey [33] or the Health and Activity Limitation Index (HALex) [34]. There were eight included studies that assessed physical function or frailty [11, 28–31, 35–37]. Physical functioning was assessed in included studies with various measures including assessment of activities of daily living (ADLs) using a 7-item scale [38] or the Older American Resources and Services activities of daily living 14-item scale [39] and objective tests of physical function such as the Short Physical Performance Battery [40]. Frailty was largely assessed using the Fried et al. [41] criteria that include self-reported weight loss, exhaustion, low grip strength, low walking speed and low physical activity. Mental health was assessed in ten included studies [18, 19, 30, 42–47] using a range of self-rating scales from the Centre for Epidemiologic Studies-Depression Scale (CES-D) [48] and Geriatric Depression Scale [49], which assess depressive symptoms, to the Hospital Anxiety and Depression Scale (HADS) [50] and the Kessler Psychological Distress Scale (K10) [51], which assess depressive and anxiety symptoms. Eighteen included studies assessed cognitive function [19, 52–68] ranging from assessment of global cognitive function using the Mini–Mental State Examination (MMSE) [69] to comprehensive evaluations of cognitive function using a battery of tests which assess a variety of cognitive functions, including short-term verbal memory, verbal and mathematical reasoning, vocabulary and verbal fluency.

### Studies using dietary indices

Table 1 describes the twenty-four studies that applied an a priori dietary index to characterise whole diet [11, 18, 27–31, 35–37, 42, 43, 52–63]. There were nine cross-sectional and fifteen longitudinal studies. The majority of studies were from European countries (*n* = 10) [11, 18, 31, 36, 37, 54, 55, 60, 62, 63], with nine from the USA or Canada [27, 35, 42, 56–59, 61], two from Australia [28, 43], two from Hong Kong [29, 53] and one from Japan [30]. Sample sizes ranged from 96 to 8660 participants. Eighteen studies included over 1000 participants [18, 27–29, 35, 36, 42, 43, 52, 53, 55–61, 63]. Dietary intake data were most commonly collected using a FFQ [11, 18, 27–30, 35, 37, 42, 43, 52–54, 56–61], followed by dietary history method [31, 36, 63], and with only one study each using multiple 24-h recalls [55] or a food diary [62].

There were twelve unique indices used in the included studies, with six studies including multiple indices [36, 52, 55, 56, 59, 61] and four studies including both indices and data-driven methods in their analysis [18, 35, 43, 53]. The majority of studies (*n* = 14) included an index, which assessed adherence to the Mediterranean diet, with the Mediterranean Diet Score (MDS) developed by Trichopoulou [8] most commonly used in nine studies [11, 31, 36, 43, 53, 55, 56, 58, 60], followed by the Med Diet Score (MedDiet) developed by Panagiotakos [70] used in three studies [42, 54, 59]. Only one study each used the Mediterranean-style dietary pattern score (MSDPS) developed by Rumawas [71], alternate Mediterranean food (MED) score developed by Fung [72] and Mediterranean Diet Adherence Screener (MEDAS) [73] developed to assess the Mediterranean diet characteristic of Spain [36, 37, 55]. One study [52] assessed adherence to a Mediterranean-style diet across eight components commonly included in the above scores, but generated scores by ranking participants according to their intake of each component and then summing the participant’s component rank scores. This study [52] also assessed adherence to the Dietary Approaches to Stop Hypertension (DASH) diet using a similar sum of eight-ranked components scoring method [74].

The remaining eight indices captured diet quality through assessment of adherence to recommended dietary guidelines or assessment of food diversity or variety. The Healthy Eating Index-2005 (HEI-2005) [75], which reflects adherence to US dietary guidelines or variations of this score, was used in four studies [27, 56, 57, 59]. The Healthy Diet Indicator (HDI) developed by Huijbregts et al. [76], which reflects adherence to World Health Organization dietary guidelines for prevention of chronic diseases, was used in two studies [62, 63]. One study [35] used the revised version of the Diet Quality Index (DQI-R) [77], which reflects US dietary guidelines, whilst another study [29] used the Diet Quality Index-International (DQI-I) [78], based on current worldwide and individual national dietary guidelines. A study examining diet quality, depression and anxiety used an a priori healthy diet quality score [18], whilst another study [28] used a modified version of the Australian diet quality index [79], based on Australian dietary guidelines. The remaining two studies [30, 61] used measures of dietary variety including the 11-item Food Diversity Score Kyoto (FDSK-11), and the recommended and non-recommended food score (RSF and NRFS [80, 81]), respectively.

There were four out of five studies that reported positive associations between a dietary index and QoL or health status [27–30]. A US study of 4009 men and women aged over 74 years reported poorer health-related QoL assessed by the HALex in participants with poor diet quality compared participants with ‘healthy’ diet quality as assessed by a dietary screening tool [27]. Woo et al. [29] found that better diet quality assessed by the DQI-I was associated with better mental and physical component summary scores from the SF-12 in 3611 older people aged 65 years or over living in Hong Kong. Another smaller study of 689 older adults aged 65 years or over from Japan found that participants that reported greater food diversity had better self-reported QoL on a visual analogue scale [30]. These cross-sectional observations were supported by a recent longitudinal study of 1305 men and women aged 55 years and over, which reported positive associations between an Australian diet quality index and physical health components of the SF-36 5 years later [28]. However, another longitudinal study and the only one of the five which used a Mediterranean diet index failed to find an association. Furthermore, a study of 216 men and 264 women from seven European countries did not find an association between diet quality assessment by the MDS and deterioration in health status of 10 years [31].

Seven out of eight studies reported positive associations between a dietary index and a measure of physical function or frailty [11, 28–30, 35–37]. A study from Germany of 192 older people aged 75 years and over found that better adherence to a Mediterranean diet was associated with reduced risk of overall frailty [37]. Another study from Spain also found associations between two Mediterranean diet indices, the MDS and the MEDAS, and frailty after a mean follow-up of 3.5 years in 1815 older adults aged 60 years and over [36]. Two other studies using the MDS developed by Trichopoulou reported mixed findings: a longitudinal cohort study of 935 women and men aged 65 years and over living in Tuscany, Italy, found that a higher score on the MDS was associated with less decline in lower body physical function over 9 years and a lower risk of developing a mobility disability [11]. In contrast, a study using the same index in a group of 216 men and 264 women aged 70–75 years across seven European countries did not find an association between the MDS and self-care ability over 10 years [31].

The remaining four studies found positive associations between diet quality and physical function or frailty outcomes. Woo et al. found that higher diet quality assessed by the DQI-I was associated with lower frailty in 3611 older men and women living in Hong Kong [29], whilst a US study found an association between better diet quality assessed by the DQI-R and lower frailty assessed by the Fried criteria after a mean follow-up of 4.6 years in 5922 older men [35]. A study of 689 older people from Japan found that participants with greater dietary variety reported better basic and advanced ADLs [30]. A longitudinal study of 895 older Australians also reported a 50 % lower risk of impaired instrumental ADLs after 5 years in the highest quartile of diet assessed by the modified Australian diet quality index compared to the lowest quartile [28].

All four studies that included mental health measures reported associations between better diet quality and lower depression [18, 30, 42, 43]. A study of 5731 Norwegian men and women aged 46–49 and 70–74 years reported that an a priori healthy diet score was inversely related to depression and anxiety in women and depression in men [18]. Food diversity was also associated with lower depression scores in 689 older Japanese [30]. Hodge et al. [43] and Skarupski et al. [42] were the only longitudinal studies to assess diet quality and mental health, with both using a Mediterranean diet index (MDS and MedDiet, respectively). Both studies reported associations between higher adherence to a Mediterranean diet and better mental health, via lower psychological distress assessed by the K10 12 years later [43] and reduced depressive symptoms after a mean of 7.2 years assessed by the CES-D [42].

There were ten out of twelve studies that reported positive associations between a dietary index and cognitive function [52, 54–57, 59–63], although some of these were reported in univariate analysis, subscales of cognitive assessments or subcohorts of the main analysis. Three studies used either the HEI-2005 [82] or a version adapted for Canadian conditions [56, 57, 59]. A study of 1269 Puerto Rican adults aged 45–75 years living in the Greater Boston area of Massachusetts found that higher scores on the HEI-2005 were related to better global cognitive function and neuropsychological tests of memory but not executive function and attention domains [56]. By including participants aged from 45 years, this study used a relatively younger population which enabled researchers to investigate possible protective factors for cognitive function from an earlier age. A study of 3790 participants aged 65 years and over from the Chicago Health and Aging Project (CHAP) [59] assessed global cognitive function over time at 3-year intervals for a mean of 7.6 years using a composite of assessments including East Boston tests of immediate and delayed recall, MMSE and Symbol Digits Modalities Test. Despite including participants of older age than the previous study, there was no association between diet quality and cognitive function. A study of 1488 Canadian adults aged 67–84 years found that the C-HEI, an adaptation of the HEI-2005 for Canadian conditions, was associated with the modified Mini–Mental Status Examination (3MS) in univariate analysis but not after adjustment for covariates [57].

Two studies used the HDI [76] developed by Huijbregts et al. and based on the WHO guidelines for prevention of chronic diseases. Huijbregts related the HDI to cognitive function measured using the MMSE in 1049 men aged 70–91 years in five cohorts from the Seven Countries Study [63]. Whilst a higher HDI score was associated with lower prevalence of cognitive impairment (defined as a MMSE ≤23) in four out of five cohorts, the associations remained significant in only one cohort after adjustment for confounders. A study of 96 free-living older people aged 64–93 years found that better cognitive performance was associated with past (1993) but not concurrent (1997) HDI [62]. This association was observed despite a generally healthy and cognitively intact sample, with participants with significant cognitive impairment excluded. A study of 3634 men and women aged ≥65 years found that participants who reported greater dietary variety as assessed by the RFS reported better cognitive function assessed by the 3MS at baseline and less decline in 3MS score over 11 years after adjusting for confounders [61]. A longitudinal study of 3580 men and women aged 65 years and over also reported an association between higher adherence to a DASH-style diet and cognitive function assessed by the 3MS after an 11 years follow-up [52].

There were eight studies that investigated the relationship between a measure of compliance with a Mediterranean diet and cognitive function. The most commonly used index was the MDS developed by Trichopoulou and colleagues [8], used by six studies [52, 53, 55, 56, 58, 60]. Significant associations between higher MDS and better performance on the MMSE were reported by Ye et al. [56] in a cross-sectional study of 1269 Puerto Rican adults aged 45–75 years from the Greater Boston area of Massachusetts and Feart et al. [60] in prospective cohort study of 1410 adults ≥65 years from Bordeaux, France, over 5 years. However, a study of 3670 men and women from Hong Kong reported no association between the MDS and cognitive function assessed using the Community Screening Instrument for Dementia (CSI-D) [53]. A study of 2504 women aged ≥65 years with pre-existing CVD or risk factors also reported no association between adherence to a Mediterranean diet assessed by the MDS and cognitive decline over 5.4 years assessed by the TICS, a telephone measure of global cognitive function based on the MMSE, or assessments of verbal memory and category fluency [58]. In contrast, a study of 3083 participants from the SU.VI.MAX study investigated associations between midlife consumption of a Mediterranean diet and cognitive performance 13 years later found associations between higher MDS and better performance on specific cognitive assessments, namely phonemic fluency and backward digit span score, but not an overall composite score built from the tests [55]. Finally, a longitudinal study of 3580 men and women aged 65 years and over reported an association between higher adherence to a Mediterranean diet and cognitive function assessed by the 3MS after an 11 years follow-up [52].

Two studies used the MedDiet score developed by Panagiotakos et al. [70] to assess adherence to a Mediterranean diet, with mixed findings. A study of 3790 adults aged ≥65 years participating in the Chicago Health and Aging Project reported positive associations between a higher MedDiet score and better cognitive function assessed by a battery of tests [59]. A study of 237 men and 320 women aged ≥65 years residing in rural Greece found a positive association between MedDiet score and MMSE score in men, but an inverse association in women [54]. The authors suggested that this unexpected relationship between diet and cognitive function in women could be due to the higher rate of cognitive impairment and lower education status observed in women compared to men in this sample, which could have confounded the association.

### Studies using data-driven methods

Table 2 describes the thirteen studies that applied a data-driven method such as cluster analysis or principal component analysis to characterise whole diet [18, 19, 43–47, 53, 64–68]. There were no studies using data-driven methods of dietary pattern assessment, which reported a measure of physical function or frailty. There were five cross-sectional and eight longitudinal studies. The majority studies were from European countries (*n* = 5), with three from the UK, two each from Australia and Hong Kong, one from Canada. Sample sizes ranged from 878 to 12,404 participants. Dietary intake data were most commonly collected by a FFQ (11 studies) [18, 19, 43–47, 53, 64, 66, 68], with two studies using multiple 24-h diet records [65, 67]. There was significant variation in the number and type of food groups used in the analysis (20–168). The number of reported factors or clusters ranged from two to six. The majority of included studies used principal component analysis (PCA) or factor analysis to derive dietary patterns [18, 43–47, 64, 66–68], whereas only one study used cluster analysis [19]. One study used the reduced rank regression (RRR) method to derive a dietary pattern associated with higher plasma levels of carotenoids [65].

One study investigated associations between data-driven dietary patterns and a measure of health status. Samieri et al. [19] investigated dietary patterns using cluster analysis in 1724 French men and women aged 65 years and over. They identified five dietary clusters for each sex, with men in the ‘paster eaters’ cluster and women in the ‘biscuits and snacking’ cluster more likely to report poor self-rated health compared to ‘healthy’ eaters. There were no other statistically significant relationships between other clusters and self-rated health, although there were relationships between clusters and other outcomes included in the study, namely cognitive function and depression, as described below.

Seven studies investigated the relationship between data-driven methods of dietary patterns and cognitive function [19, 53, 64–68]. Six reported a relationship between a ‘healthy/prudent’ [19, 64, 67], ‘traditional’ [66], ‘vegetable–fruits’ [53], ‘snacks–drinks–milk products’ pattern [53] or ‘whole food’ [68] pattern and better cognitive function, despite using varying measures of cognitive function. One study reported a relationship between a ‘Mediterranean-style’ dietary pattern and better cognitive function [66]. Two studies reported a relationship between a ‘processed food/Western’ pattern and poorer cognitive function [64, 68]. The relationship was observed in both cross-sectional and longitudinal studies (with up to 13 years follow-up), which provides further support for a possible relationship between dietary patterns and cognitive function in older people. One study of 2983 men and women investigated the association between a carotenoid-rich dietary pattern and cognitive performance 13 years later [65]. They used RRR to derive a dietary pattern associated with higher levels of plasma carotenoids at baseline, which was characterised by consumption of orange- and green-coloured fruits and vegetables, vegetable oils and soup and was found to be associated with better cognitive function assessed by a battery of tests.

Seven studies investigated the relationship between a measure of mental health and data-driven dietary patterns [18, 19, 43–47]. The majority of studies reported relationships between a ‘healthy’, ‘whole food’, ‘vegetables–fruits’ or ‘Mediterranean’ diet and better mental health [18, 44–47], with some studies also reporting a relationship between an ‘unhealthy’, ‘processed food’ or ‘western’ diet and poorer mental health [46, 47]. Samieri et al. [19] investigated the cross-sectional relationship between dietary clusters and depressive symptoms using the CES-D in 1724 French older adults. They identified five dietary clusters in each sex and found men in the ‘paster eaters’ cluster had higher depressive symptoms than participants in the ‘healthy’ cluster. Rienks et al. [45] also investigated the relationship between depressive symptoms using the CES-D with dietary patterns derived by PCA in women aged 50–55 years. Whilst they identified six dietary patterns, only higher consumption of the ‘Mediterranean-style diet’ pattern was associated with lower depressive symptoms at baseline and after 3 years, and no other relationships with patterns were reported after adjustment for covariates. Similarly, consumption of a ‘whole food’ dietary pattern characterised by high consumption of vegetables, fruits and fish was associated with lower odds of depression (measured by CES-D), in a study of 3486 adults from the Whitehall II prospective cohort [47]. This study also found a negative association between a ‘processed food’ pattern and depression. Le Port et al. [46] also identified five dietary patterns in men and six in women using PCA in a sample of 9272 men and 3132 women aged 45–60 years at baseline. They reported relationships between depressive symptoms over 10 years using the CES-D and multiple dietary patterns in their cohort, including lower likelihood of depressive symptoms in the ‘healthy’ pattern in men and women and ‘traditional’ pattern in women only, and increased likelihood of depression in the ‘low fat’ and ‘snacking’ patterns in men and women, and ‘western’ and ‘fat-sweet’ patterns in men only.

Jacka et al. [18] investigated the association between dietary patterns and depression and anxiety assessed by the Hospital Anxiety and Depression Scale (HADS) in 5731 Norwegian adults aged 46–49 and 70–74 years. They identified three dietary patterns using PCA and found that a ‘traditional Norwegian’ dietary pattern was associated with lower depression scores in men and lower anxiety scores in women, a ‘healthy’ dietary pattern was associated with lower depression and anxiety scores in women but increased anxiety scores in men. Hodge et al. [43] also investigated the relationship between dietary patterns derived from PCA and psychological distress incorporating depression and anxiety using the K10 distress survey 12 years later in 8660 men and women aged 50–69 years at baseline. They identified two dietary patterns, a ‘modified Mediterranean’ pattern and an ‘Australian-style’ pattern, which included some foods high in fat and sugar content along with whole foods. Surprisingly, the authors reported no relationship between a ‘modified Mediterranean’ pattern and mental health, but a significant weak inverse association between an ‘Australian-style’ pattern and a lower K10 score at follow-up. The authors concluded that the significant association with the ‘Australian-style’ pattern could be attributed to a ‘community belonging’ factor, rather than the specific foods or nutrients provided, although several measures of social support were included in the model as covariates. Chan et al. [44] also reported unexpected associations between dietary patterns derived from factor analysis and depressive symptoms measured using the Geriatric Depression Scale in 2902 adults aged 65 years and over. They identified three patterns, the ‘vegetable–fruits’, ‘snacks–drinks–milk products’ and ‘meat–fish’ patterns and reported associations between consumption of the ‘vegetable–fruits’ pattern and lower depressive symptoms at baseline, but no associations with any dietary pattern after a 4 years follow-up. Surprisingly, they also reported an association between lower depressive symptoms at baseline and consumption of the ‘snacks–drinks–milk products’ pattern, which was composed of a mixture of healthy foods including wholegrains and milk and unhealthy foods including sweets, fast food and French fries.

### Risk of bias assessment

Overall, three (9 %) studies included had a low risk of bias, 24 (71 %) had a moderate risk of bias and seven (20 %) had a high risk of bias (Online Resource Table 1). There were 24 (71 %) studies that reported the number of and/or reasons for dropouts and withdrawals. Many studies included references to other studies containing reliability information for the dietary intake and outcome measures they reported, and for others this information was readily available in the literature. Six (17 %) studies included measures with no reliability information available. Two studies provided no information on validity of dietary intake or outcome measures, and this information could not be found in the literature.

## Discussion

Overall, this first systematic review of dietary patterns and successful ageing supports a relationship between dietary intake assessed by whole-diet approaches and measures of cognitive function and mental health in older people. For QoL and physical function, the number of studies is small with too few longitudinal studies to draw strong conclusions, although initial findings are suggestive of a relationship between a healthier diet and better health outcomes. Although advances in health care have resulted in extended life expectancy, it is now important that the onset of chronic disease and illness is delayed and older people are able maximise their QoL, health and independence during this time. In essence, health expectancy needs to be extended in companion with life expectancy. To date, reviews investigating relationships between dietary patterns and health in older people have focussed on mortality [83] and chronic disease outcomes such as cardiovascular disease [84] and diabetes [85]. This review adopted a broader approach by including health status, physical function and mental health outcomes, which are risk factors for poorer morbidity and mortality outcomes overall in older age [86].

This review provides support that dietary indices or patterns are related to health status and QoL in older people. The majority of studies that included a measure of health status or QoL reported a positive relationship between a healthy or better quality diet which reflected current dietary recommendations and better outcomes in older people. To our knowledge, this is the first systematic review of dietary patterns, health status and QoL in older people. We found five studies of health status or QoL measures, which used an a priori index of diet quality of adherence to a diet, and one which used a posteriori or empirically derived dietary patterns. Despite the broadly consistent findings, there is heterogeneity across whole-diet assessment methods and health status/QoL outcomes used in these previous studies. It should also be noted that only two studies have investigated the relationship between dietary patterns and QoL longitudinally [28, 31], with inconsistent findings. Further longitudinal research into the association and comparing associations across different whole-diet assessment methods within samples will help interpretation of this body of research in the future.

Referring to physical function and frailty, this review provided support for a relationship between whole-diet assessment measures and these outcomes longitudinally in older people. There have been no published reviews of the literature of dietary patterns and frailty or physical function to date. There were seven out of eight included studies that reported a relationship between an a priori index of diet quality or adherence to a particular diet and better physical function or reduced frailty risk using report-based and objective measures in older adults. Previously, diet quality indices have been associated with lower intakes of energy, total fat and saturated fat and higher intakes of fibre, β-carotene, vitamin C, folate, calcium and iron [7] and also biomarkers of chronic disease risk including serum homocysteine, serum C-reactive protein, plasma glucose, total serum cholesterol and blood pressure [87]. These dietary indices may therefore represent adherence to a diet associated with maintenance of muscle strength and reduced likelihood of disability and chronic disease in this study [88], a key requirement for QoL and physical function in older age [89]. No included studies investigated the relationship between a posteriori or empirically derived dietary patterns and physical function, and this should be investigated in future studies.

Of the studies in this current review, cognitive function was the most commonly assessed outcome. A number of recent reviews have also investigated whole-diet assessment methods and cognitive function [90, 91], although these were not systematic reviews. That a large amount of dietary patterns work in this age group has focussed on cognitive function outcomes is perhaps to be expected, considering the importance of this outcome in older age. Dementia is one of the most commonly reported neuropsychological conditions in older adults [92] and the fourth leading cause of death among high-income countries [93]. Although the evidence has been broadly supportive of a relationship between a ‘healthy’ diet and better cognitive function or reduced risk of decline, there are some inconsistencies among results, particularly in Mediterranean diet adherence indices.

There has been growing interest in the relationship between dietary patterns and mental health across a wide range of sample populations [94, 95]. This is the first review of dietary patterns to focus on mental health in older age. Our review is consistent with previous reviews in children and younger adults [94, 95], which concluded that there is generally a consistent relationship between diet quality or a ‘healthy’ diet and better mental health. However, there are some inconsistencies in the literature to date and a reliance on cross-sectional studies, resulting in concerns about possible reverse causality (i.e. depressive symptoms encouraging poor dietary habits) in these samples, and therefore, further research in this area is needed [96].

There are a number of proposed mechanisms by which a healthy diet as assessed by dietary index or pattern may support mental health and cognitive function. The brain requires nutrients for development of its structure and function from birth, and this requirement continues through the lifespan [97]. For example, a healthy diet such as the Mediterranean-style diet including high consumption of vegetables, fruits, legumes, olive oil, fish, cereals, nuts and seeds can provide a range of nutrients including B vitamins, omega-3 fatty acids and antioxidants [98]. Antioxidants can protect the brain against oxidative damage to cellular membranes, which has been implicated in psychiatric disorders including depression [99]. The omega-3 fatty acid docosahexaenoic acid is highly concentrated in the structure of the brain and critical for brain development. Omega-3 fatty acids and vitamins also influence a variety of brain functions including production of neurotransmitters, neuronal cell growth and survival and protection of the blood brain barrier [98].

Despite the popularity of indices which measure adherence to a Mediterranean-type diet to date, there were more positive associations observed in studies using dietary guidelines or variety indices than Mediterranean diet indices. This may partially reflect the difficulty in adapting this score for non-Mediterranean populations. For example, the MED score by Fung et al. [72] was adapted for use in a non-Mediterranean population, which may make it more suitable for use in non-Mediterranean populations than other Mediterranean diet scores. This may explain why positive associations were seen with a measure of frailty [37], compared to the original MDS which reported positive associations with frailty with a sample of older Italians [11], but not a mixed sample across seven European countries [31]. These mixed findings reflect the complex nature and variations of dietary patterns across samples, and again comparison of whole-of-diet analysis methods within samples may provide further insight.

More of the studies included in this review used a priori indices, rather than a posteriori or empirical approaches. The popularity of a priori indices in previous research may be explained partly due to the variety of scores available for use, combined with their relative ease of use and interpretation compared to a posteriori approaches. Although some other systematic reviews have found more studies using dietary indices than empirical approaches in young children [100], this is not the case in adults [101]. Although both approaches use similar dietary intake information such as diet records or FFQs, the final dietary pattern provided by each approach and the conclusions which can be drawn vary significantly. The a priori approach can determine whether a sample population is meeting a diet previously defined by healthy eating recommendations and can be used and compared across study samples. In contrast, the a posteriori approach reflects the current dietary profiles within the sample and is unique to the specific sample, although considerable reproducibility across populations has been demonstrated [102]. Of the studies that included an a posteriori approach to diet assessment, more studies derived patterns using a PCA or factor analysis rather than cluster analysis, which also reflects previous reviews [6]. Ultimately, both approaches can provide valuable information on the relationship between dietary intake and health outcomes.

The assessment of multiple components of dietary intake within the one measure is also a strength of the dietary pattern or whole-of-diet analysis approach. For example, Huijbregts et al. [63] investigated the relationship between HDI and cognitive function across five cohorts from three countries. The dietary components of the HDI that were associated with cognitive function were not the same in each of the cohorts. This supports the hypothesis that the combination of different dietary components—the dietary pattern—is responsible for observed association with cognitive function. The dietary pattern approach, and particularly the use of dietary indices, also allows dietary intake to be captured and compared across different cohorts or samples whilst allowing for variation in components across different cohorts or samples. However, it is possible that lack of variation in reported dietary intake in some cohorts could reduce variation in dietary indices and contribute to lack of statistically significant associations observed in some studies, as proposed by Huijbregts et al. [63].

As there is considerable variation in dietary intake and dietary pattern analysis methods, so is there also considerable variation in methods of assessment of QoL, physical function, mental health and cognitive function our outcome measures of interest. Cognitive function had the most varied assessment, from broad assessment of global cognitive function to neuropsychological assessment of specific functions, with variation in reported results across outcomes. For example, significant associations between higher MDS and better performance on the MMSE as a measure of global cognitive function were reported by Ye et al. [56] and Feart et al. [60]. However, no significant associations were observed with neuropsychological test batteries, which assessed specific cognitive functions separately, highlighting the importance of selection of assessments to measure cognitive functions of interest likely to be affected by diet. Measures used to assess physical function and frailty also varied from self-report of ability to complete ADLs to objective assessment via a battery of physical performance tasks. Associations with dietary patterns were reported across these varying measures, providing further strength to the reported relationships. Health status and QoL were assessed using standard self-report measures, which varied from a single-item measure to the more detailed multiple-item measures such as the SF-36. Significant associations with dietary patterns were observed across the range of QoL measures used. Studies investigating mental health also used a range of high-quality self-report measures widely used in epidemiological studies and reported associations across a wide range of measures. Therefore, it appears that choice of appropriate assessment method may be most crucial to interpretation of studies investigating cognitive function.

Limitations of the studies included in this review included the dietary assessment measures used, the large number of cross-sectional studies, particularly for QoL and mental health outcomes, and issues relating to bias due to non-response and loss to follow-up. The risk of bias was high with only three studies deemed to have a low risk of bias. Assessment of dietary intake is challenging, and the majority of studies used FFQ measures of dietary intake which have known levels of under-reporting and bias [103]. This represents an ongoing limitation of the nutrition literature as a whole, and there are ongoing efforts to improve dietary intake assessment methods [104, 105]. Although some studies did assess diet at multiple time-points, the majority used only one assessment of dietary intake and could not examine longitudinal changes in dietary intake. It is possible the studies may include selective non-response, where people with the poorest health status may not participate or be able to complete questionnaires and therefore reduce variation in health outcome reported, which might be particularly relevant for cognitive function and mental health outcomes. A large proportion of the studies were cross-sectional, particularly in QoL and mental health studies, which represent a lower level of evidence than longitudinal as causality cannot be determined and reverse causality is possible. In contrast, a higher proportion of studies investigating cognitive function and physical function were longitudinal, perhaps reflecting a recognition of the importance of measuring decline in these outcomes over time in research to date. There was substantial heterogeneity in the studies, reflected in multiple measures of dietary intake, whole-diet assessment techniques and assessment of health outcome measures, which meant that the studies could not be pooled even within outcome type. Although a comprehensive search strategy was employed, publication bias through reduced likelihood of publication of studies with negative findings cannot be ruled out.

This review adds to previous evidence by indicating that diet quality and is linked to health expectancy in addition to life expectancy [83] and chronic disease [106]. A strength of this review is the focus on population-based and community-based samples of older adults. It is generally accepted that finding ‘true’ associations between lifestyle factors such as diet and health outcomes requires a large healthy population at baseline [107]. The exclusion of papers focusing on clinical or residential care based populations helps to meet this condition for the current review, in addition to the focus of samples aged 45 years and over which is commonly when chronic disease starts to appear. Other strengths include the comprehensive search strategy with eight databases systematically searched supplemented by hand searching and the inclusion of multiple approaches of dietary pattern assessment.

Further research is required in this area, particularly investigating associations between dietary patterns and physical function and health-related QoL measures. Physical function should be a particular focus, considering its importance as a predictor of future disability, residential care and mortality [108–110]. Further research should also investigate the use of different whole-of-diet assessment methods and how the choice of dietary index or statistical measure may influence the results. Another area for future research that has emerged from this review is the difference in associations between dietary patterns and health outcomes observed between men and women in many of the studies, particularly in mental health and cognitive function outcomes [18, 19, 54]. The underlying reasons for these differences are not clear. It is plausible that response biases in dietary reporting and health outcomes could have contributed to the difference in men and women observed in the included studies. There are also differences in prevalence of reported mental health conditions between men and women, with women more likely to suffer from depression and anxiety [111, 112]. However, there is evidence that depressive symptoms increase at a greater rate over time in older men, and gender differences in severity disappear completely in the years preceding death [113]. Therefore, further investigation of mental health and dietary patterns in older populations over time is warranted.

## Conclusion

This systematic review fills a substantial gap in the literature about dietary patterns and successful ageing. The findings demonstrate evidence of an association between a better quality or a healthy diet and better health-related QoL, physical function and mental health in older age. There have been limited studies to date, and more longitudinal studies are needed, particularly in QoL and health status outcomes. Given the promising findings, there is a need for further high-quality research in this area.

## Electronic supplementary material

Supplementary material 1 (DOCX 24 kb)
