# Supplementary material for: Dietary patterns and successful ageing: a systematic review
Source: Eur J Nutr. 2015 Dec 22;55:423–50. doi: 10.1007/s00394-015-1123-7 (PMC4767865; doi:10.1007/s00394-015-1123-7)
Supplement: Supplementary file 1 — Supplementary material 1 (DOCX 24 kb) [file 394_2015_1123_MOESM1_ESM.docx]

Dietary patterns and successful ageing: a systematic review

**European Journal of Nutrition**

Catherine M Milte* and Sarah A McNaughton

*Centre for Physical Activity and Nutrition Research, School of Exercise and Nutrition Sciences, Deakin University, Melbourne, Victoria, Australia*

*Corresponding author: Dr Catherine Milte

Centre for Physical Activity and Nutrition Research

School of Exercise and Nutrition Sciences

Deakin University

221 Burwood Highway

Burwood

Vic 3125

AUSTRALIA

Ph: +61 3 92445416

Fax: +61 3 92446017

Email: [catherine.milte@deakin.edu.au](mailto:catherine.milte@deakin.edu.au)

**Online Resource Table 1** Risk of bias assessment

| Author (year) | Selection bias | Study design | Confound | Blinding | Data collection | Withdrawals and dropouts | Global risk of bias* |
| --- | --- | --- | --- | --- | --- | --- | --- |
| Akbaraly et al. (2009)[1] | Mod | Mod | Low | Mod | Low | High | Mod |
| Akbaraly et al. (2009)[2] | Mod | Mod | Low | Mod | Low | Mod | Mod |
| Bollwein et al. (2013)[3] | High | Mod | High | Mod | Mod | Low | High |
| Chan et al. (2014)[5] | Mod | Mod | Low | Mod | Low | Low | Mod |
| Chan et al. (2013)[4] | Mod | Mod | Low | Mod | Low | Low | Mod |
| Corley et al. (2013)[6] | High | Mod | Mod | Mod | Low | High | High |
| Feart et al. (2009)[7] | High | Mod | Low | Mod | Low | Mod | Mod |
| Ford et al. (2014)[8] | Mod | Mod | Low | Mod | Low | High | Mod |
| Gopinath et al. (2014)[9] | Low | Mod | Low | Mod | Mod | Mod | Mod |
| Haveman-Nies et al. (2003)[10] | Mod | Mod | High | Mod | High | High | High |
| Hodge et al. (2013)[11] | Mod | Mod | Low | Mod | Low | Mod | Mod |
| Huijbregts et al. (1998)[12] | High | Mod | Low | Mod | Low | Low | Mod |
| Jacka et al. (2011)[13] | Mod | Mod | Low | Mod | Low | Low | Mod |
| Katsiardanis et al. (2013)[14] | Mod | Mod | Low | Mod | Low | Low | Mod |
| Kesse-Guyot et al. (2014)[15] | Mod | Mod | Low | Low | Mod | High | Mod |
| Kesse-Guyot et al. (2013)[17] | High | Mod | Low | Mod | Low | High | High |
| Kesse-Guyot et al. (2012)[16] | High | Mod | Low | Mod | Low | Low | Mod |
| Kimura et al. (2009)[18] | High | Mod | High | Mod | Low | High | High |
| Le Port et al. (2012)[19] | High | Mod | Low | Mod | Low | Low | Mod |
| Leon-Munoz et al. (2014)[20] | Mod | Mod | Low | Mod | Mod | Low | Mod |
| Milaneschi et al. (2011)[21] | Low | Mod | Low | Mod | Low | High | Mod |
| Nicolas et al. (2000)[22] | High | Mod | High | Mod | Low | High | High |
| Parrott et al. (2013)[23] | Mod | Mod | Low | Mod | Low | Low | Mod |
| Rienks et al. (2013)[24] | Low | Mod | Low | Mod | Low | Low | Low |
| Samieri et al. (2008)[25] | Low | Mod | Low | Mod | Low | Low | Low |
| Shatenstein et al. (2012)[26] | Mod | Mod | Low | Mod | Low | Low | Mod |
| Shikany et al. (2014)[27] | Mod | Mod | Low | Mod | Low | Low | Mod |
| Skarupski et al. (2013)[28] | Mod | Mod | Low | Mod | Low | High | Mod |
| Tangney et al. (2011)[29] | Mod | Mod | Low | Mod | Low | Mod | Mod |
| Vercambre et al. (2012)[30] | High | Mod | Low | Mod | Low | Low | Mod |
| Wengreen et al. (2013)[31] | Low | Mod | Low | Mod | Low | Low | Low |
| Wengreen et al. (2009)[32] | Low | Mod | Low | Mod | Low | Mod | Mod |
| Woo et al. (2010)[33] | High | Mod | High | Mod | Low | Low | High |
| Ye et al. (2013)[34] | High | Mod | Low | Mod | Low | Low | Mod |

Global risk of bias ratings defined as: ‘low’ (four low ratings and no high ratings) ‘medium’ (less than four low ratings and one high rating), ‘high’ (two or more high ratings from the six components).

References

1. Akbaraly TN, Brunner EJ, Ferrie JE, Marmot MG, Kivimaki M, Singh-Manoux A (2009) Dietary pattern and depressive symptoms in middle age. Br J Psychiatry 195:408-413.

2. Akbaraly TN, Singh-Manoux A, Marmot MG, Brunner EJ (2009) Education attenuates the association between dietary patterns and cognition. Dement Geriatr Cogn Disord 27:147-154.

3. Bollwein J, Diekmann R, Kaiser MJ, Bauer JM, Uter W, Sieber CC, Volkert D (2013) Dietary Quality Is Related to Frailty in Community-Dwelling Older Adults. J Gerontol A Biol Sci Med Sci 68:483-489.

4. Chan R, Chan D, Woo J (2013) A cross sectional study to examine the association between dietary patterns and cognitive impairment in older Chinese people in Hong Kong. J Nutr Health Aging 17:757-765.

5. Chan R, Chan D, Woo J (2014) A prospective cohort study to examine the association between dietary patterns and depressive symptoms in older Chinese people in Hong Kong. PLoS ONE 9:e105760-e105760.

6. Corley J, Starr JM, McNeill G, Deary IJ (2013) Do dietary patterns influence cognitive function in old age? Int Psychogeriatr 25:1393-1407.

7. Feart C, Samieri C, Rondeau V, Amieva H, Portet F, Dartigues JF, Scarmeas N, Barberger-Gateau P (2009) Adherence to a Mediterranean diet, cognitive decline, and risk of dementia. JAMA 302:638-648.

8. Ford D, Jensen G, Still C, Wood C, Mitchell D, Erickson P, Bailey R, Smiciklas-Wright H, Coffman D, Hartman T (2014) The associations between diet quality, body mass index (BMI) and health and activity limitation index (HALex) in the Geisinger Rural Aging Study (GRAS). J Nutr Health Aging 18:167-170.

9. Gopinath B, Russell J, Flood VM, Burlutsky G, Mitchell P (2014) Adherence to dietary guidelines positively affects quality of life and functional status of older adults. J Acad Nutr Diet 114:220-229.

10. Haveman-Nies A, De Groot LCPGM, Van Staveren WA (2003) Relation of dietary quality, physical activity, and smoking habits to 10-year changes in health status in older Europeans in the SENECA study. Am J Public Health 93:318-323.

11. Hodge A, Almeida OP, English DR, Giles GG, Flicker L (2013) Patterns of dietary intake and psychological distress in older Australians: benefits not just from a Mediterranean diet. Int Psychogeriatr 25:456-466.

12. Huijbregts PPCW, Feskens EJM (1998) Dietary patterns and cognitive function in elderly men in Finland, Italy and the Netherlands. Eur J Clin Nutr 52:826.

13. Jacka FN, Mykletun A, Berk M, Bjelland I, Tell GS (2011) The association between habitual diet quality and the common mental disorders in community-dwelling adults: the Hordaland Health Study. Psychosom Med 73:483-490.

14. Katsiardanis K, Diamantaras A-A, Dessypris N, Michelakos T, Anastasiou A, Katsiardani K-P, Kanavidis P, Papadopoulos FC, Stefanadis C, Panagiotakos DB, Petridou ET (2013) Cognitive Impairment and Dietary Habits Among Elders: The Velestino Study. J Med Food 16:343-350.

15. Kesse-Guyot E, Andreeva VA, Ducros V, Jeandel C, Julia C, Hercberg S, Galan P (2014) Carotenoid-rich dietary patterns during midlife and subsequent cognitive function. Br J Nutr 111:915-923.

16. Kesse-Guyot E, Andreeva VA, Jeandel C, Ferry M, Hercberg S, Galan P (2012) A healthy dietary pattern at midlife is associated with subsequent cognitive performance. J Nutr 142:909-915.

17. Kesse-Guyot E, Andreeva VA, Lassale C, Ferry M, Jeandel C, Hercberg S, Galan P (2013) Mediterranean diet and cognitive function: a French study. Am J Clin Nutr 97:369-376.

18. Kimura Y, Wada T, Ishine M, Ishimoto Y, Kasahara Y, Konno A, Nakatsuka M, Sakamoto R, Okumiya K, Fujisawa M, Otsuka K, Matsubayashi K (2009) Food diversity is closely associated with activities of daily living, depression, and quality of life in community-dwelling elderly people. J Am Geriatr Soc 57:922-924.

19. Le Port A, Gueguen A, Kesse-Guyot E, Melchior M, Lemogne C, Nabi H, Goldberg M, Zins M, Czernichow S (2012) Association between Dietary Patterns and Depressive Symptoms Over Time: A 10-Year Follow-Up Study of the GAZEL Cohort. PLoS ONE 7.

20. Leon-Munoz LM, Guallar-Castillon P, Lopez-Garcia E, Rodriguez-Artalejo F (2014) Mediterranean Diet and Risk of Frailty in Community-Dwelling Older Adults. J Am Med Direct Assoc 15:899-903.

21. Milaneschi Y, Bandinelli S, Corsi AM, Lauretani F, Paolisso G, Dominguez LJ, Semba RD, Tanaka T, Abbatecola AM, Talegawkar SA, Guralnik JM, Ferrucci L (2011) Mediterranean diet and mobility decline in older persons. Exp Gerontol 46:303-308.

22. Nicolas AS, Faisant C, Nourhashemi F, Lanzmann-Petithory D, Vellas B (2000) Associations between nutritional intake and cognitive function in a healthy ageing sample: A 4-year reassessment. European Journal of Geriatrics 2:114-119.

23. Parrott MD, Shatenstein B, Ferland G, Payette H, Morais JA, Belleville S, Kergoat MJ, Gaudreau P, Greenwood CE (2013) Relationship between diet quality and cognition depends on socioeconomic position in healthy older adults. J Nutr 143:1767-1773.

24. Rienks J, Dobson AJ, Mishra GD (2013) Mediterranean dietary pattern and prevalence and incidence of depressive symptoms in mid-aged women: results from a large community-based prospective study. Eur J Clin Nutr 67:75-82.

25. Samieri C, Jutand M, Féart C, Capuron L, Letenneur L, Barberger-Gateau P (2008) Dietary patterns derived by hybrid clustering method in older people: association with cognition, mood, and self-rated health. J Am Diet Assoc 108:1461-1471.

26. Shatenstein B, Ferland G, Belleville S, Gray-Donald K, Kergoat MJ, Morais J, Gaudreau P, Payette H, Greenwood C (2012) Diet quality and cognition among older adults from the NuAge study. Exp Gerontol 47:353-360.

27. Shikany JM, Barrett-Connor E, Ensrud KE, Cawthon PM, Lewis CE, Dam T-TL, Shannon J, Redden DT (2014) Macronutrients, diet quality, and frailty in older men. J Gerontol A Biol Sci Med Sci 69:695-701.

28. Skarupski KA, Tangney CC, Li H, Evans DA, Morris MC (2013) Mediterranean diet and depressive symptoms among older adults over time. J Nutr Health Aging 17:441-445.

29. Tangney CC, Kwasny MJ, Li H, Wilson RS, Evans DA, Morris MC (2011) Adherence to a Mediterranean-type dietary pattern and cognitive decline in a community population. Am J Clin Nutr 93:601-607.

30. Vercambre MN, Grodstein F, Berr C, Kang JH (2012) Mediterranean diet and cognitive decline in women with cardiovascular disease or risk factors. J Acad Nutr Diet 112:816-823.

31. Wengreen H, Munger RG, Cutler A, Quach A, Bowles A, Corcoran C, Tschanz JT, Norton MC, Welsh-Bohmer KA (2013) Prospective study of Dietary Approaches to Stop Hypertension- and Mediterranean-style dietary patterns and age-related cognitive change: the Cache County Study on Memory, Health and Aging. Am J Clin Nutr 98:1263-1271.

32. Wengreen HJ, Neilson C, Munger R, Corcoran C (2009) Diet quality is associated with better cognitive test performance among aging men and women. J Nutr 139:1944-1949.

33. Woo J, Chan R, Leung J, Wong M (2010) Relative contributions of geographic, socioeconomic, and lifestyle factors to quality of life, frailty, and mortality in elderly. PLoS ONE 5.

34. Ye X, Scott T, Gao X, Maras JE, Bakun PJ, Tucker KL (2013) Mediterranean diet, healthy eating index 2005, and cognitive function in middle-aged and older puerto rican adults. J Acad Nutr Diet 113:276-281.e273.
